# Letter from London

**Published:** 1884-08

**Authors:** 


### CORRESPONDENCE.

LONDON, ENGLAND, June 30, 1884.

EDITOR DENTAL REGISTER:

Thinking a few notes on dental topics from this side might not prove uninteresting to your readers, I will endeavor to give you the result of observations as succinctly as possible.

While traveling through England I availed myself of opportunities that presented themselves of coming in contact with the representative men of the places at which I might be stopping, and endeavored to secure an exchange of ideas concerning our chosen profession.

I found the practice of dentistry in England only to be contrasted with that of America, the mode of recognized practice in both countries diverging from its incipiency; here the tendency is to degenerate into a misanthropic state, speaking in a general manner, owing to a tendency, or rather a prevailing custom of having a sliding scale of fees, which is in no wise governed by the extent of the operation performed or service rendered; such a state of affairs necessarily precludes the possibility of a man performing his operations in a conscientious manner, or of taking the interest in his patients that he would did a different state of affairs exist.

But possibly this state of affairs is brought about by the indifference and inattention of the British public to the welfare of their dental organs, for it is well known that they would not tolerate the protracted operations we are all conversant with, but are easily satisfied with a temporary operation taking but a few minutes for its completion, preferring to visit their dentists two or three times a year to have the operation repeated, and *finally* resort to the inevitable, extraction, than to having a thorough operation performed at once; but right here I think the dentist is to blame, for if he was the right sort of a man he would not so far forget himself and sink his identity so low as to cater to such a depraved condition of affairs. And as far as ethics are concerned, the millenium of the English dentist is in the dim distant future.

The materials used for the preservation of the natural teeth consist almost exclusively of amalgams of various degrees of vileness, and plastics, of which they have a variety, most of them being quite good; Fletcher's preparations being considered about the best. Gold is but little used, its manipulation being but little understood here. One dentist I met here said "he would like to spend an hour or two and learn how to make such stoppings," referring to some gold fillings I had shown him.

The manipulation of plastic fillings is quite well understood here, but the application of rubber dam for the perfect attainment of results being almost entirely ignored I can hardly understand how they meet with the success that they do. Most operators here depend upon hand excavation for the preparation of the cavity for the reception of the filling, dental engines being used by a very limited number.

In prosthetic dentistry they can claim considerable proficiency in so far as metal plates are concerned, many displaying ingenuity, combined with a constructive capacity surpassed by few if by any; but for vulcanite and celluloid work I can say but little of a commendatory nature. I have seen no gum teeth of English manufacture here in any offices I have been in, but must say that some of their plain teeth are the acme of perfection, they possessing a studied artistic rendition of nature that is pleasant in the extreme, combined as it is both in contour and shading.

One line of practice I have noticed that to me seems of a very disgusting type, *i. e.*, the insertion of artificial teeth over old decayed roots that are the cause of hypertrophied and apthous gums and discharging abscesses; such a course of procedure seems as irrational as it must be deadly; for the systemic conditions produced by the entailed pollution of the portal of nutrition cannot help but make itself manifest by a general depression of the patient's vitality.

While in Paris I visited quite a number of the best and most successful practitioners, who were nearly all American dentists, there I found some that had not entirely forgotten the teachings of their preceptors, and that by their zealous endeavors were trying to place dentistry upon the pedestal they wished it to occupy, but sorry to say they were in an insignificant minority; for there I also found the same state of affairs existing as described in speaking of England, the practice in general being identical in the two countries.

Dental education seems to be the topic that is most thorougly discussed in the societies of both countries, and advancement in the science and art of dentistry must necessarily follow, but its evolution must necessarily be slow, for with the present laws, that tolerate the veriest charlatan, they having been in practice before said laws became established facts; it is time alone that will obliterate the uncouth ideas disseminated by parasites of that description.

I must say that the hospitality extended me by dentists both in England and France has been of the heartist description and I found them, as a rule, enjoying their practices, be they large or small, and enjoying life as well as any class of citizens, but sorry to say that I did not find the intelligent earnest dentist occupying the social distinction here that he does in the United States.

